# Systematic analysis of gene expression alterations and clinical outcomes of STAT3 in cancer

**DOI:** 10.18632/oncotarget.23226

**Published:** 2017-12-14

**Authors:** Xiangrong Cui, Xuan Jing, Qin Yi, Chunlan Long, Bin Tan, Xin Li, Xueni Chen, Yue Huang, Zhongping Xiang, Jie Tian, Jing Zhu

**Affiliations:** ^1^ Pediatric Research Institute, Children’s Hospital of Chongqing Medical University, Ministry of Education Key Laboratory of Child Development and Disorders, Chongqing 400014, China; ^2^ China International Science and Technology Cooperation Base of Child Development and Critical Disorders, Chongqing 400014, China; ^3^ Chongqing Key Laboratory of Pediatrics, Chongqing 400014, China; ^4^ Cardiovascular Department (Internal Medicine), Children’s Hospital of Chongqing Medical University, Chongqing 400014, China; ^5^ Clinical laboratory, Shanxi Province people’s hospital, Shanxi 030000, Taiyuan, China

**Keywords:** STAT3, overall survival, mutation, copy number alteration

## Abstract

Accumulated studies have provided controversial evidences of prognostic value for signal transducer and activator of transcription proteins 3 (STAT3) in cancers. To address this inconsistency, we performed a systematic analysis to determine whether STAT3 can serve as a prognostic marker in human cancers. STAT3 expression was assessed using Oncomine analysis. cBioPortal, Kaplan-Meier Plotter, and Prognoscan were performed to identify the prognostic roles of STAT3 in human cancers. The copy number alteration, mutation, interactive analysis, and visualize the altered networks were performed by cBioPortal. We found that STAT3 was more frequently overexpressed in lung, ovarian, gastric, blood and brain cancers than their normal tissues and its expression might be negatively related with the prognosis. In addition, STAT3 mutation mainly occurred in uterine cancer and existed in a hotspot in SH2 domain. Those findings suggest that STAT3 might serve as a diagnostic and therapeutic target for certain types of cancer, such as lung, ovarian, gastric, blood and brain cancers. However, future research is required to validate our findings and thus promote the clinical utility of STAT3 in those cancers prognosis evaluation.

## INTRODUCTION

Cancer is one of the major causes threatening human health and life [1]. Despite significant advances in diagnostic and treatment modalities, the average five-year survival rate for cancer patients is still extremely poor [2]. Stepwise accumulation of somatic genetic alterations is the basis for cancer which involved in base insertions, deletions, substitutions, translocation events, and copy number alteration [3–5]. The fact that targeted therapy has been successful in part of cancers calls for a better comprehension of the pathological mechanisms responsible for these oncogenic alterations leading to cancer.

Signal transducer and activator of transcription proteins 3 (STAT3), a member of STAT family, is well demonstrated to exerts an important effect on tumorigenesis and tumor-related inflammation [6]. Aberrant expression and persistent activation of STAT3 is implicated in cell proliferation, differentiation, apoptosis and immune escape, inducing and maintaining a pro-carcinogenic inflammatory microenvironment [7]. Continuously activated STAT3 has been found in many human tumors, such as lung cancer [8], gastric carcinoma [9], cervical carcinoma [10], and meningiomas [11]. Moreover, mounting evidence have demonstrated STAT3-targeted therapy could effectively inhibit tumor development in various human cancers [12]. However, the prognostic value of STAT3 overexpression in human tumors is still controversial. Therefore, in the current study, we carried out a systematic analysis combining thousands of gene expression or copy number variation analysis published online, to evaluate the expression pattern, potential functions and distinct prognostic value in cancer of STAT3.

## RESULTS

**Figure 1:**
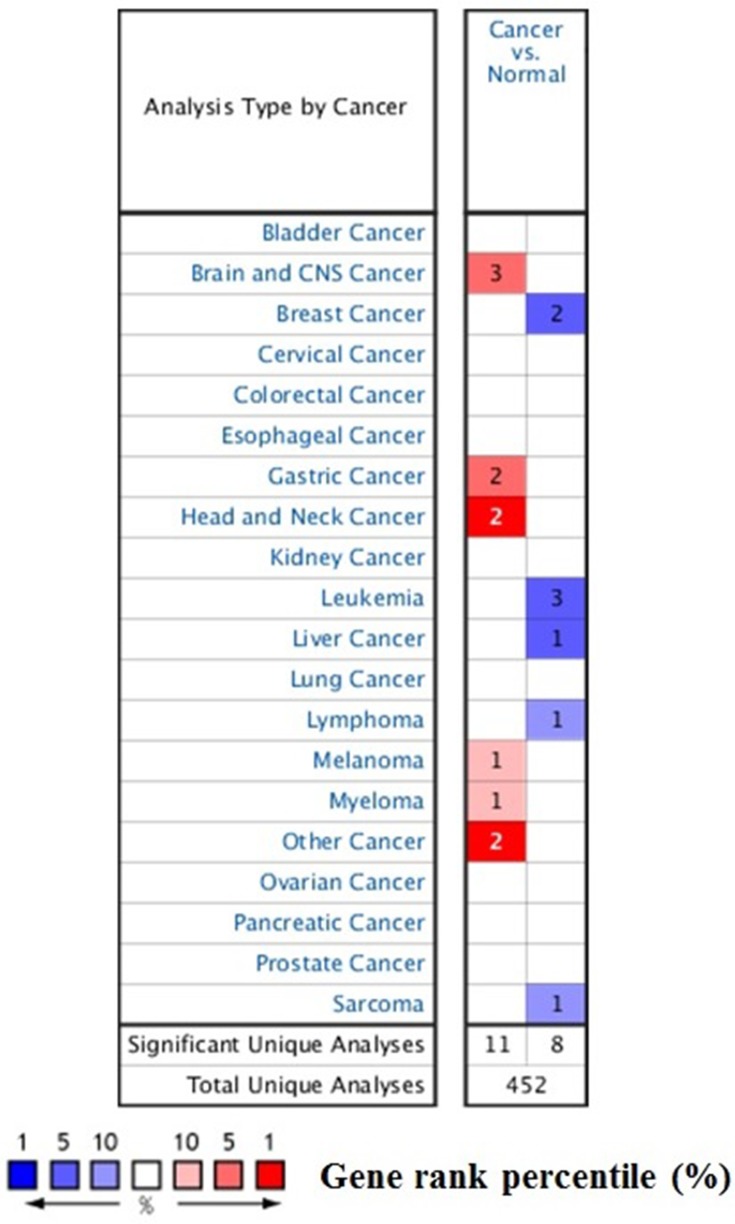
The transcription levels of STAT3 in different types of cancers This graphic was generated from Oncomine, indicating the numbers of datasets with statistically significant (*p* < 0.01) mRNA over-expression (Red) or down-expression (Blue) of STAT3 (different types of cancer vs. corresponding normal tissue). The threshold was designed with following parameters: *p*-value of 1E-4, fold change of 2, and gene ranking of 10%.

To explore the role of STAT3 in cancers, we compared the transcription levels of STAT3 in cancers with that in normal tissues, using Oncomine database and found that the mRNA expressions of STAT3 were significantly over-expressed in certain types of tumors and lower in others as compared to that of the normal sample. As show in Figure 1, STAT3 may work either oncogenic or anti-oncogenic function based on the cancer types. Therefore, detailed analyses of STAT3 were described below.

### The transcript expression of STAT3 in different cancer types

**Figure 2:**
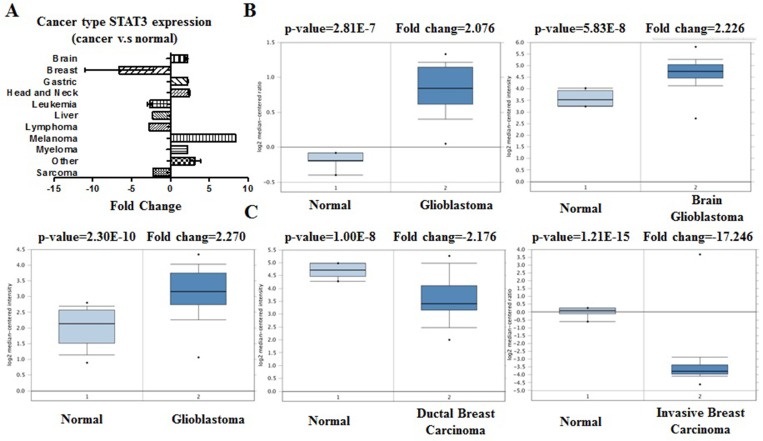
STAT3 analysis in different cancer types (Oncomine database) The box plot comparing specific STAT3 expression in normal (left plot) and cancer tissue (right plot) was derived from Oncomine database. The fold change of STAT3 in various types of cancers was identified from our analyses in Table 1 and expressed as the forest plot (**A**). The analysis was shown in glioblastoma carcinoma relative to normal breast (**B**), in breast carcinoma relative to normal pancreatic (**C**).

We conducted cDNA microarray analysis by using the Oncomine database to explore gene expression of STAT3 in cancer types. The Oncomine database was queried for STAT3 expression in cancer and normal tissues. Our analysis revealed that STAT3 was over-expressed in brain and cns, gastric, head and neck, melanoma, myeloma cancers, but was under-expressed in breast, leukemia, liver, lymphoma, and sarcoma cancers as compared to that in normal tissue (Table 1, Figure 2A–2C. Supplementary Figures 1–3) [13–28]. These observations are in agreement with the previously published reports on STAT3 expression [29]. For instance, our study indicated that STAT3 is highly expressed in glioblastoma (Figure 2B) [29], elevated in hepatocellular cancer (Supplementary Figure 2B) [30].

**Table 1:** STAT3 expression in cancers

Cancer	Cancer subtype	*p*-value	Fold change	Rank (%)	Sample	Reference
**Brain**	Glioblastoma	2.81E-7	2.076	4	31	[13]
	Glioblastoma	2.30E-10	2.270	7	104	[14]
**Breast**	Ductal Breast Carcinoma	1.00E-8	–2.176	2	47	[15]
	Invasive Breast Carcinoma Stroma	1.21E-15	–11.013	10	59	[16]
**Gastric**	Gastric Mixed Adenocarcinoma	6.45E-6	2.190	3	35	[17]
	Gastric Intestinal Type Adenocarcinoma	2.26E-10	2.252	3	57	[17]
**Head and Neck**	Salivary Gland Adenoid Cystic Carcinoma	7.94E-8	2.560	2	22	[18]
	Tongue Carcinoma	1.81E-7	2.284	1	37	[19]
**Leukemia**	B-Cell Acute Lymphoblastic Leukemia	3.69E-41	–2.179	3	221	[20]
	B-Cell Acute Lymphoblastic Leukemia	1.76E-10	–2.669	5	93	[21]
	T-Cell Acute Lymphoblastic Leukemia	1.86E-6	–3.149	7	17	[21]
**Liver**	Hepatocellular Carcinoma	4.66E-9	–2.290	3	57	[22]
**Lymphoma**	Burkitt’s Lymphoma	9.06E-5	–2.746	8	42	[23]
**Melanoma**	Cutaneous Melanoma	8.09E-5	8.442	10	52	[59]
**Myeloma**	Monoclonal Gammopathy of Undetermined Significance	1.14E-5	2.213	7	66	[25]
**Other**	Teratoma, NOS	1.34E-10	2.490	1	20	[26]
	Pleural Malignant Mesothelioma	6.05E-5	3.884	4	49	[27]
**Sarcoma**	Myxoid/Round Cell Liposarcoma	2.12E-5	–2.229	9	29	[28]

### Genetic alterations of STAT3 and overall survival (OS)

**Figure 3:**
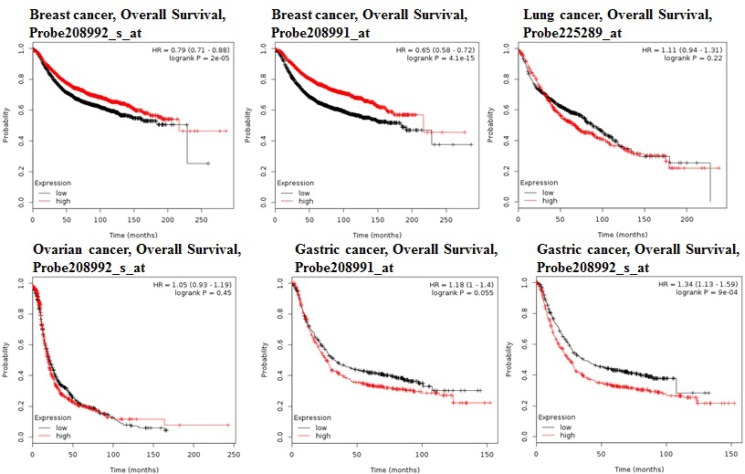
STAT3 genes in Breast, Ovarian, Gastric and Lung cancer (Kaplan–Meier Plotter) The survival curve comparing the patient with high (red) and low (black) expression in breast, ovarian, gastric and lung cancer was plotted from KaplanMeier plotter database.

Using the comprehensive Kaplan-Meier survival analysis platform, we discovered that decreased mRNA expression of STAT3 is an unfavorable prognostic factor of overall survival for patients with breast adenocarcinoma (Figure 3). Contrastingly, lung, ovarian, and gastric cancers showed the relationship between overexpression of STAT3 and overall low survival rates (Figure 3).

**Figure 4:**
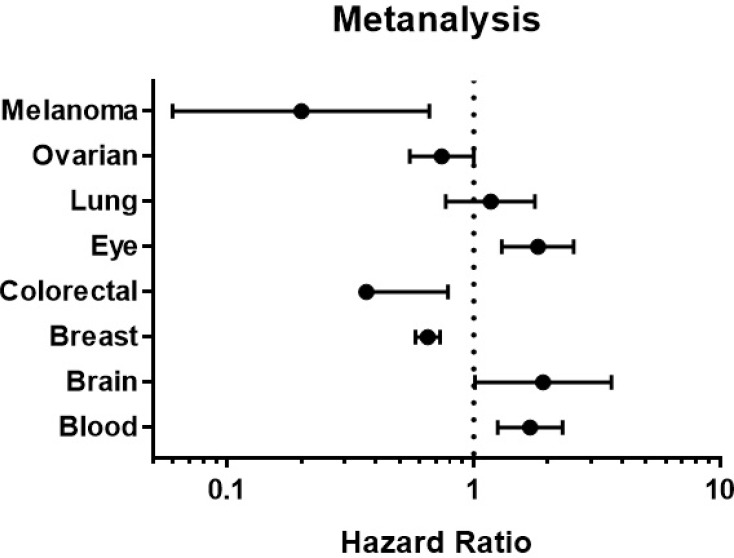
STAT3 genes in different cancer types (PrognoScan database) The statistically significant hazard ratio in various types of cancers was identified from our analyses in Table 2 and expressed as the forest plot. The analysis of survival curve was identified as the threshold of cox *p*-value < 0.05.

**Figure 5:**
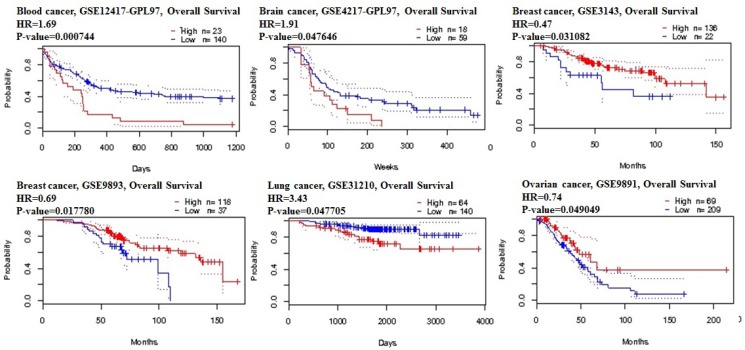
STAT3 genes in blood, brain, lung and ovarian cancer types (PrognoScan database) The survival curve comparing the patient with high (red) and low (black) expression was plotted from PrognoScan database. The survival curve comparing the patient with high (red) and low (black) expression in blood cancer, brain cancer, lung cancer and ovarian cancer was plotted from PrognoScan database as the threshold of cox *p*-value < 0.05.

The prognostic value of STAT3 expression was reported by PrognoScan database (Figure 4, Table 2). The poor prognosis in ovarian cancer patients with higher STAT3 expression (Figure 5) was in line with the data from Kaplan–Meier plotter analysis (Figure 3). Using the comprehensive survival analysis platforms Kaplan-Meier plotter, Oncomine, and PrognoScan, we have demonstrated the oncogenic role of STAT3 in ovarian, lung, blood, and brain cancer, however, which is not clear in breast cancer.

**Table 2:** The association of STAT3 expression and the survival in cancer patients

Cancer type	Dataset	Endpoint	Probe id	*N*	Cox *p*-value	Hr
**Blood**	GSE12417-GPL97	Overall Survival	243213_at	163	0.000744	1.69
**Brain**	GSE4271-GPL97	Overall Survival	243213_at	77	0.047646	1.91
**Breast**	GSE3143	Overall Survival	39708_at	158	0.031082	0.47
	GSE7849	Disease Free Survival	39708_at	76	0.017328	4.53
	GSE7849	Disease Free Survival	289_at	76	0.014144	4.92
	GSE12276	Relapse Free Survival	208991_at	204	0.067027	0.75
	GSE6532-GPL570	Relapse Free Survival	208991_at	87	0.015187	0.54
	GSE6532-GPL570	Distant Metastasis Free Survival	208992_s_at	87	0.012369	0.49
	GSE6532-GPL570	Distant Metastasis Free Survival	208991_at	87	0.015187	0.54
	GSE6532-GPL570	Relapse Free Survival	225289_at	87	0.046274	0.52
	GSE6532-GPL570	Distant Metastasis Free Survival	225289_at	87	0.046274	0.52
	GSE6532-GPL570	Relapse Free Survival	208992_s_at	87	0.012369	0.49
	GSE9195	Relapse Free Survival	208991_at	77	0.021717	3.80
	GSE9195	Relapse Free Survival	243213_at	77	0.018195	8.75
	GSE9195	Distant Metastasis Free Survival	243213_at	77	0.035491	9.33
	GSE12093	Distant Metastasis Free Survival	208992_s_at	136	0.016456	0.59
	GSE12093	Distant Metastasis Free Survival	208991_at	136	0.005146	0.27
	GSE11121	Distant Metastasis Free Survival	208992_s_at	200	0.020347	0.44
	GSE9893	Overall Survival	21668	155	0.017780	0.69
	GSE2034	Distant Metastasis Free Survival	208992_s_at	286	0.015785	0.78
	GSE2034	Distant Metastasis Free Survival	208991_at	286	0.003215	0.53
	GSE2990	Relapse Free Survival	208991_at	62	0.037864	0.55
	GSE2990	Relapse Free Survival	208992_s_at	62	0.010824	0.33
**Colorectal**	GSE17537	Disease Free Survival	225289_at	55	0.027763	0.21
**Eye**	GSE22138	Distant Metastasis Free Survival	208992_s_at	63	0.005425	2.00
	GSE22138	Distant Metastasis Free Survival	208991_at	63	0.035231	1.66
**Lung**	GSE31210	Relapse Free Survival	208992_s_at	204	0.001112	4.53
	GSE31210	Relapse Free Survival	225289_at	204	0.017076	4.72
	GSE31210	Relapse Free Survival	243213_at	204	0.034308	0.54
	GSE31210	Overall Survival	208992_s_at	204	0.047705	3.43
	GSE8894	Relapse Free Survival	243213_at	138	0.002854	0.02
**Ovarian**	GSE9891	Overall Survival	208991_at	278	0.049049	0.74
**Skin**	GSE19234	Overall Survival	208991_at	38	0.008049	0.20

### Protein components of nodes across the STAT3

**Figure 6:**
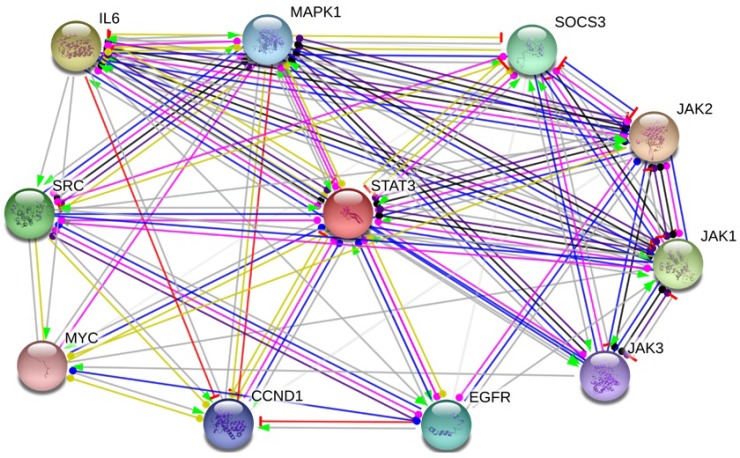
Identifcation of known and predicted structural proteins essential for STAT3 function Interacting nodes are displayed in colored circles using String, v10.0. Predicted functional partners of STAT3 are shown based upon peer reviewed published data and curated database entries. [STRING v.10 (http://string-db.org)].

STAT homologs in mammals are comprised of six conserved structural domains, as follows: N-domain (ND), coiled-coil, DNA binding, linker, Src homology 2 (SH2), and transcriptional activation domain [31, 32]. Under normal physiological conditions, the activation of STATs is strictly regulated, which could regulate cell proliferation, survival and other critical cellular functions by modulating the expression of specific target genes. In cancer, by contrast, STAT protein, especially STAT3, become activated constitutively, thereby driving the malignant phenotype of cancer cells. We selected the functional protein partners of STAT3 based on previous publications and curated databases [6, 33–37]. Hence, the following nine predicted proteins, including cyclin D1 (CCND1), epidermal growth factor receptor (EGFR), Interleukin-6 (IL6), Janus kinase 1 (JAK1), Janus kinase 3 (JAK3), mitogen-activated protein kinase 1 (MAPK1), myelocytomatosis oncogene (MYC), suppressor of cytokine signaling3 (SOCS3), SRC were chose for further analysis of STAT3 (Figure 6).

### Unbiased cross cancer subtypes relationships by cBioPortal data

**Figure 7:**
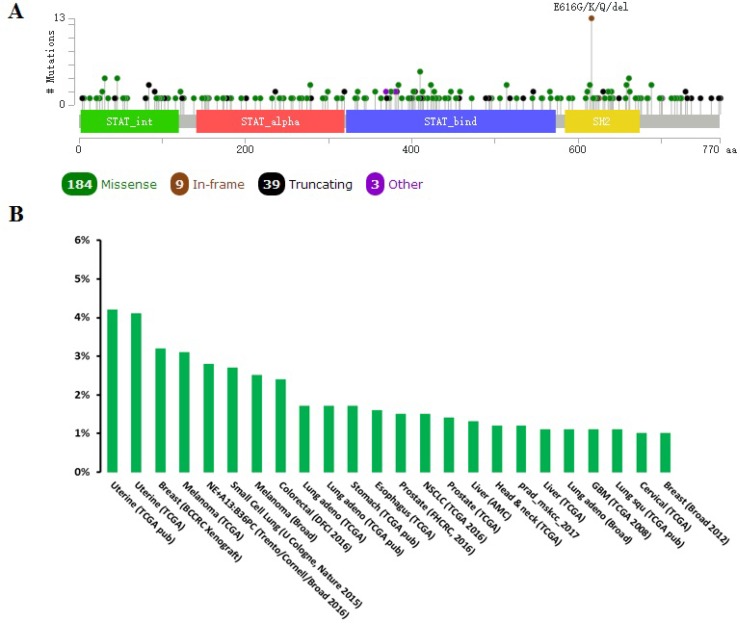
Mutation diagram of STAT3 in different cancer types across protein domains A total of 300 mutation sites were detected and located between amino acids 0 and 770. STAT3 mutation mainly occurred in uterine cancer and existed in a hotspot in SH2 domain.

**Figure 8:**
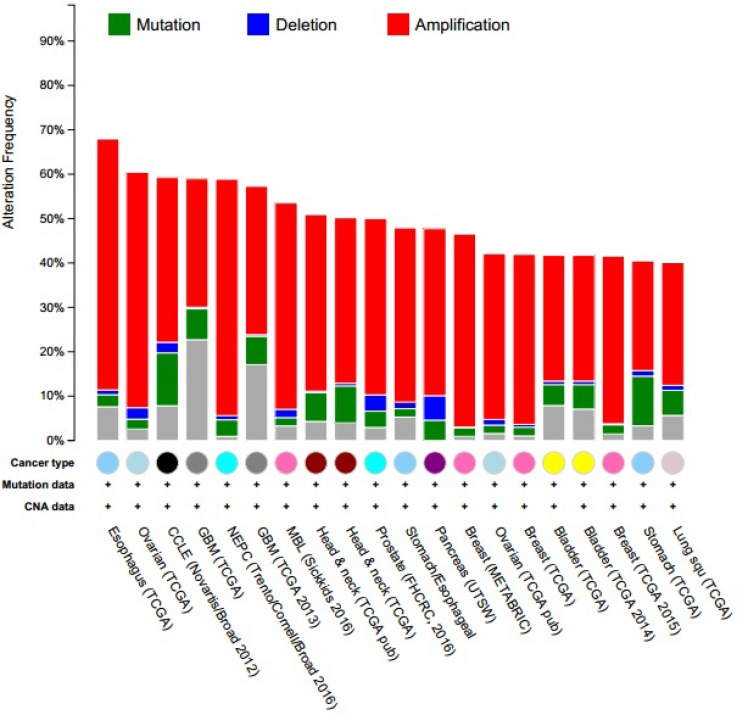
Copy number alteration of STAT3 genes and cancer subtypes The alteration frequency of a ten-gene signature (CCND1, EGFR, IL6, JAK1, JAK3, MAPK1, MYC, SOCS3, SRC, STAT3) was determined using the cBioPortal (http://www.cbioportal.org). Only cancer types containing >100 samples and an alteration frequency of >40% are shown. The alteration frequency included deletions (blue), amplifcation (red), multiple alterations (grey) or mutation (green). The total number of samples for each cancer type are indicated by the numbers at the top of each column.

When compared with the high frequency of STAT3 genetic deletions, there were few, frequent STAT3 gene mutations in 87 studies examined using cBioPortal Web. As show in Figure 7, a total of 300 mutation sites were detected and located between amino acids 0 and 770. STAT3 mutation mainly occurred in uterine cancer and existed in a hotspot in SH2 domain. In addition, we used cBioPortal tool to analyze the 10 gene of mutations and CNAs with 87 different cancer studies. The results analyzed 20 different cancer studies representing 8513 samples that contained >40% alteration frequency and at least 100 samples in the dataset (Figure 8, Table 3). From the lowest to highest dominance hierarchy, the ratio of alteration ranged over 40.1–67.9%. The particular interest constituted the predominant pattern of amplification occurring in neuroendocrine prostate cancer (NEPC).

**Table 3:** Cross-cancer alteration summary for CCND1, EGFR, IL6, JAK1, JAK3, MAPK1, MYC, SOCS3, SRC, STAT3

Cancer	Data source	*N*	Frequency (%)	Multiple alterations (%, *N*)	Amplification (%, *N*)	Mutation (%, *N*)	Deletion (%, *N*)
Esophagus	TCGA	184	67.9%	7.6% (14)	56.5% (104)	2.7% (5)	1.1% (2)
Ovarian	TCGA	311	60.5%	2.6% (8)	53.1% (165)	2.3% (7)	2.6% (8)
CCLE	Novartis/Broad 2012	881	59.3%	7.8% (69)	37.1% (327)	11.9% (105)	2.4% (21)
GBM	TCGA	273	59%	22.7% (62)	28.9% (79)	7% (19)	0.4% (1)
NEPC	Trento/Cornell/Broad 2016	107	58.9%	0.9% (1)	53.3% (57)	3.7% (4)	0.9% (1)
Glioblastoma	TCGA 2013	281	57.3%	117.1% (48)	33.5% (94)	6.4% (18)	0.4% (1)
MBL	Sickkids 2016	213	53.3%	3.3% (7)	46.5% (99)	1.9% (4)	1.9% (4)
Head & neck	TCGA pub	279	50.9%	4.3% (12)	39.8% (111)	4.3% (12)	0.4% (1)
Head & neck	TCGA	504	50.2%	4% (20)	37.3% (188)	8.3% (42)	0.6% (3)
Prostate	FHCRC, 2016	136	50%	2.9% (4)	39.7% (54)	3.7% (5)	3.7% (5)
Stomach/Esophageal	TCGA	265	47.9%	5.3% (14)	39.2% (104)	1.9% (5)	1.5% (4)
Pancreas	USTW	109	47.7%	0% (0)	37.6% (41)	4.6% (5)	5.5% (6)
Breast	METABRIC	2051	46.5%	0.9% (19)	43.5% (892)	2% (41)	0.1% (2)
Ovarian	TCGA pub	316	42.1%	1.6% (5)	37.3% (118)	1.9% (6)	1.3% (4)
Breast	TCGA	963	42%	1% (10)	38l3% (369)	2% (19)	0.6% (6)
Bladder	TCGA	127	41.7%	7.9% (10)	28.3% (36)	4.7% (6)	0.8% (1)
Bladder	TCGA	127	41.7%	7.1% (9)	28.3% (36)	5.5% (7)	0.8% (1)
Breast	TCGA 2015	816	41.5%	1.5% (12)	37.7% (308)	2.1% (17)	0.2% (2)
Stomach	TCGA	393	40.5%	3.3% (13)	24.7% (97)	11.2% (44)	1.3% (5)
Lung squ	TCGA	177	40.1%	5.6% (10)	27.7% (49)	5.6% (10)	1.1% (2)

**Figure 9:**
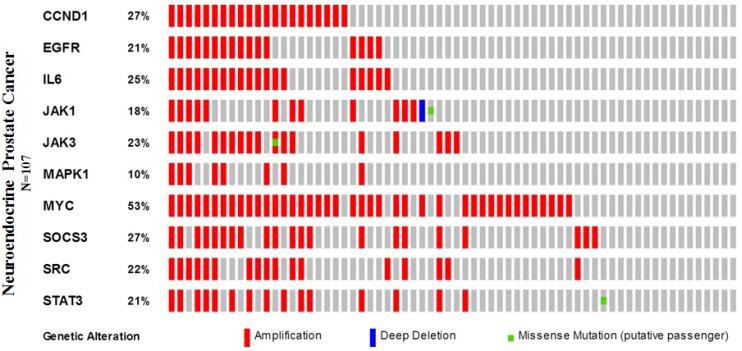
Neuroendocrine prostate cancer types frequently amplify STAT3 We used the Oncoprint feature of the cBioPortal (http://www.cbioportal.org) to determine the copy number alteration frequency of each individual gene (CCND1, EGFR, IL6, JAK1, JAK3, MAPK1, MYC, SOCS3, SRC, and STAT3) in STAT3 within selected cancer subtypes.

Furthermore, we applied the OncoPrint from a query for alterations in CCND1, EGFR, IL6, JAK1, JAK3, MAPK1, MYC, SOCS3, SRC, and STAT3 genes. The percentages of alterations in these genes among NEPC varied from 10–53% for individual genes (CCND1, 27%; EGFR, 21%; IL6, 25%; JAK1, 18%; JAK3, 23%; MAPK1, 10%; MYC, 53%; SOCS3, 27%; SRC, 22%; and STAT3, 21%), the MYC gene was amplified predominantly in the NEPC type (Figure 9, Table 4).

**Table 4:** The percentages of alterations in CCND1, EGFR, IL6, JAK1, JAK3, MAPK1, MYC, SOCS3, SRC, STAT3

Cancer	CCND1	EGFR	IL6	JAK1	JAK3	MAPK1	MYC	SOCS3	SRC	STAT3
Esophagus	36%	16%	9%	3%	5%	1.6%	27%	3%	3%	4%
Ovarian	8%	3%	4%	4%	11%	5%	41%	6%	4%	1.9%
CCLE	12%	15%	7%	8%	5%	7%	24%	8%	5%	6%
GBM	0.4%	55%	0.7%	1.1%	1.5%	1.8%	1.8%	0.4%	0.7%	1.1%
NEPC	27%	21%	25%	18%	23%	10%	53%	27%	22%	21%
Glioblastoma	0.4%	53%	0.7%	1.1%	1.1%	1.4%	1.8%	0.7%	0.7%	0.7%
MBL	25%	8%	2.8%	4%	2.8%	1.9%	19%	6%	5%	2.3%
Head & neck	28%	14%	1.4%	1.8%	1.8%	4%	13%	0.4%	1.8%	1.4%
Head & neck	25%	14%	2.2%	2.4%	2.2%	4%	13%	0.2	1.8%	1.6%
Prostate	13%	4%	6%	6%	6%	1.9%	41%	1.9%	7%	7%
Stomach/Esophageal	12%	10%	6%	2.3%	5%	1.9%	23%	4%	5%	5%
Pancreas	9%	1.8%	0.9%	6%	8%	7%	13%	10%	6%	6%
Breast	17%	4%	2.8%	3%	1.9%	1.1%	27%	6%	2.7%	1.4%
Ovarian	4%	2.2%	1.9%	1.6%	6%	2.2%	31%	2.8%	1.6%	0.6%
Breast	16%	2.7%	1.9%	2.6%	2.4%	1%	22%	6%	2.5%	2.6
Bladder	13%	11%	6%	5%	2.4%	4%	12%	4%	3%	1.6%
Bladder	13%	9%	6%	5%	3%	4%	12%	4%	3%	1.6%
Breast	16%	2.6%	1.5%	2.7%	2.6%	1.3%	21%	6%	2.8%	3%
Stomach	8%	10%	3%	6%	3%	2.3%	15%	2%	4%	2.8
Lung squ	12%	10%	5%	3%	4%	5%	10%	4%	2.3%	2.8%

**Figure 10:**
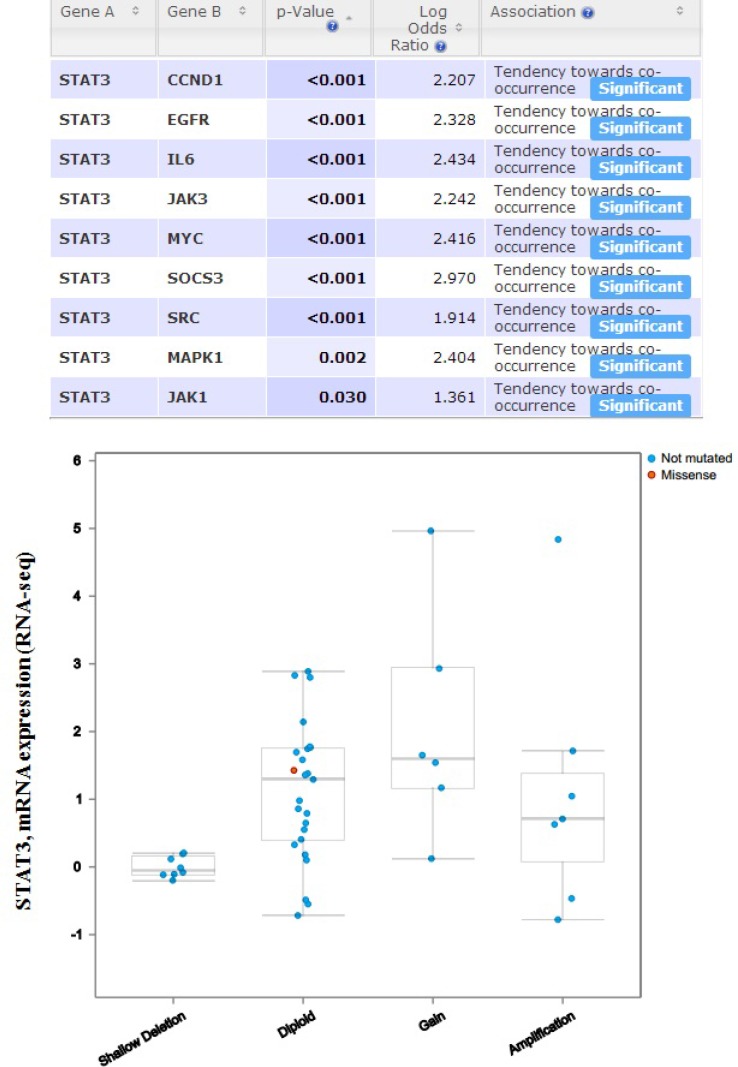
The mutual exclusivity panel analysis revealed that the co-occurrent alternations of STAT3 and CCND1, EGFR, IL6, JAK1, JAK3, MAPK1, MYC, SOCS3, SRC has statistically significant The *P* values are determined by a Fisher’s exact test, *P* < 0.05 (http://www.cbioportal.org/index.do?session_id=59847ef8498e5df2e2937e6b&show_samples=false&).

In order to discover whether each gene pair has a significant correlation, the portal performs a Fisher’s exact test. The mutual exclusivity panel analysis revealed that the co-occurrent alternations of STAT3 and CCND1, EGFR, IL6, JAK1, JAK3, MAPK1, MYC, SOCS3, SRC has statistically significant. Functional plotting of the corresponding mRNA level associated with the genetic status of STAT3 revealed that deletion of STAT3 was associated with increased mRNA expression (Figure 10).

**Figure 11:**
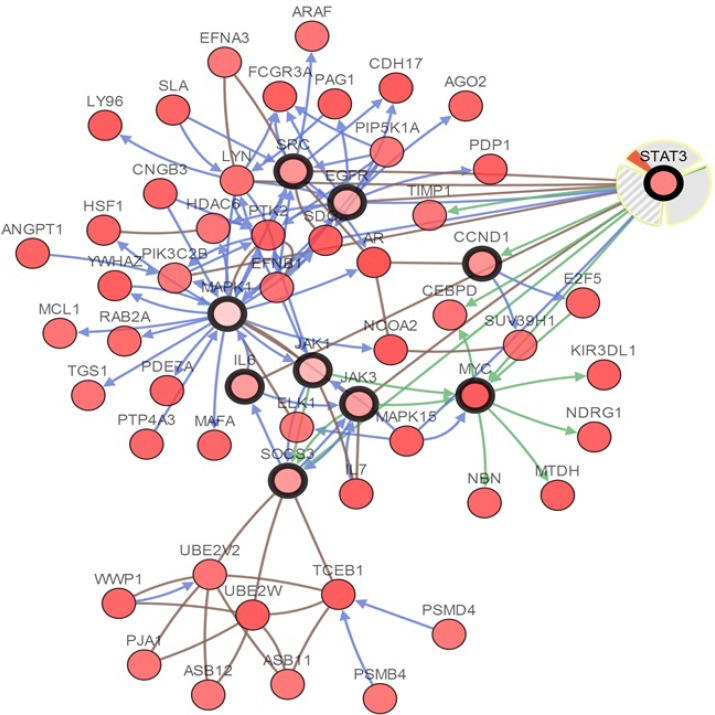
Interactive analysis and visualize the altered networks of STAT3 (cBio Cancer Genomics Portal) Darker red indicates increased frequency of alteration (defned by mutation, copy number amplifcation, or homozygous deletion) in Neuroendocrine Prostate Cancer.

The cBioPortal analysis program identified 12 types of human cancer with significant CNAs in the chosen genes’ signature (STAT3, CCND1, EGFR, IL6, JAK1, JAK3, MAPK1, MYC, SOCS3, and SRC). The STAT3 signature was created such as to represent the structures and functions of STAT3. The CNAs of specific structural components of the STAT3 in tumors may be potential targets to prevent metastatic spread. Network view of STAT3 and other chosen genes in neuroendocrine prostate cancer was presented in Figure 11. The query genes, STAT3, CCND1, EGFR, IL6, JAK1, JAK3, MAPK1, MYC, SOCS3, and SRC were depicted with a thick border and neighbor genes were distributing around them.

## DISCUSSION

STAT3 has been proved to participate in the generation and development of various cancers [38]. Moreover, numbers of researches have shown that STAT3-targetd therapy can effectively inhibit tumor development [12]. However, the exact role of STAT3 overexpression in human tumors is till controversial. In order to have the compelling analysis, in the current research, we performed the analyses depend on numerous genes expression with clearly defined parameters between cancer and normal tissues. In Oncomine analysis, STAT3 was found to be unregulated in brain and CNS, gastric, head and neck, melanoma, myeloma cancer, but deregulated in breast, leukemia, liver, lymphoma, and sarcoma cancer.

To gain further insights into the role of a prognostic marker, we next investigated the association of STAT3 expression and OS in various cancers, the prognostic value of STAT3 mRNA expression was assessed using the Kaplan-Meier Plotter and PrognoScan. Overall, high levels of STAT3 gene expression result in low survival in ovarian, lung, blood, and brain cancer, however, which is not clear in breast cancer. Therefore, to assess the tumorigenic or tumor suppressor role of STAT3 in breast cancer, many previous studies have demonstrated that the protein expression was significantly up-regulated in breast cancer tissues compared with their matched normal breast tissues [39–41]. Furthermore, the expression of STAT3 in tumor tissues was significantly associated with a tumor, lymph node metastasis, and TNM stage in breast patients [42]. In addition, Kaplan-Meier analysis demonstrated that the overall survial rate in breast cancer patients with high STAT3 levels was significantly lower than that in those with low STAT3 levels [41].

Somatically acquired genetic, epigenetic, transcriptomic, and proteomic alternations are the major four factors in tumorigenesis [4]. The somatic loss-of-function or gain-of-function alterations are happened in specific genomic regions, which could indicate their potential inhibitory or carcinogenic roles, respectively [43]. Therefore, we used cBioPortal to identify human cancers discovered significant CAN in the STAT3-gene signature. STAT3 mutation mainly occurred in uterine cancer and existed in a hotspot in SH2 domain. From the lowest to highest dominance hierarchy, the ratio of alteration ranged over 40.1–67.9%. The particularly interest constituted the predominant pattern of amplification occurring in neuroendocrine prostate cancer (NEPC).

Subsequently, we performed cBioPortal to interactive analysis and visualize the altered networks of STAT3. From the network analysis, we can discover more information about the mechanisms of interaction among the different genes [5]. Figure 11 displayed that the Network view of the STAT3 neighborhood in neuroendocrine prostate cancer, those results were better to comprehend the molecular mechanisms of STAT3 underlying cancer. After an extensive literature review on previous related studies, STAT3 is proven to involve in various tumors by impacting target genes or signal pathway, which is consistent with our bioinformatics analyses [6, 44–49]. As the previous studies revealed that STAT3 transactivates proliferative genes (*cMyc* and *CyclinD1*), prosurvival genes (*Bcl-xl* and *Survivin*) and invasive genes (*VEG-f* and *Klf-8*), leading to fast-growing tumors with highly metastatic capability [50]. Yuanyan Li *et al.* recently demonstrated that BMX can promote cell proliferation through STAT3 signaling pathways in cervical cancer cells [51], meanwhile Zhongde Zhang *et al.* revealed that STAT3 could bind promoter region of TXNDC17 for regulating its expression and mediating Taxol resistance via enhancing autophagy in human colorectal cancer cells [52]. Along with the mechanistic insights, identification of the cell context-dependent functions for STAT3 may help ultimately develop therapeutic strategies targeting STAT3.

In the present study, we used portals to systemically analyze the expression and prognostic value of STAT3 in cancer development, which contributes to a better understanding of molecular etiology and epidemiology of cancer, and ultimately accelerates the transformation of genomic knowledge into clinical practice. Our finding demonstrates that STAT3 might serve as a diagnostic and therapeutic target for certain types of cancer, including lung, ovarian, gastric, blood and brain cancers. However, the deep mechanism of these results remains unclear, further researches need to be performed in the future.

## MATERIALS AND METHODS

### Oncomine database analysis

Oncomine database (https://www.oncomine.org/resource/login.html), an online database consisting of previously published and open-access microarray data, was performed to identify the transcription level of STAT3 gene in various types of cancers [53, 54]. The mRNA expression of STAT3 in clinical cancer tissue was compared with that in normal control, using a Students’ *t*-test to generate a *p* value. The parameters *p*-value < IE-4, fold change >2, and gene ranking in the top 10% were used to obtain the most significant STAT3 probes. Heat map was used to define the co-expression profiles of STAT3 gene in different types of cancers.

### cBioPortal database analysis

The cBioPortal for Cancer genomics is an open-access resource (http://www.cbioportal.org/) [55, 56], providing visualization and analyzing tool for more than 5,000 tumor samples from 105 cancer studies in TCGA pipeline. The search interface combined with customized data storage enabled researchers to interactively explore genetic alterations across samples from other cancer studies and specific genes. The term “STAT3” was searched in cBioPortal database and a cross-cancer summary was obtained for it. The search parameters included alterations (amplification, deep deletion, missense mutations), copy-number variance (CNV) from GISTIC and RNA seq data with the default setting. OS and DFS were calculated on the basis of cBioPortal’s online instruction.

### Kaplan–Meier plotter database analysis

Kaplan-Meier Plotter (http://kmplot.com/analysis/) is an online database of published microarray datasets that assess the effect of 54,675 genes on survival using 10,461 cancer samples (5,143breast, 1,816 ovarian, 2,437 lungs and 1,065 gastric cancer) [57]. We performed the Kaplan-Meier plotter to assess the prognostic value of STAT3 expression in patients with breast, gastric, ovarian and lung cancer. The hazard ratio (HR) with 95% confidence intervals (CI) and log rank *p*-value was also computed.

### Prognoscan database analysis

PrognoScan (http://www.prognoscan.org/) is a comprehensive online platform for assessing potential tumor biomarkers and therapeutic targets. We used the PrognoScan platform to validate the prognostic value of STAT3 expression in patients with various types of cancers. The threshold was adjusted to cox *p*-value < 0.05.

### Identifying the protein components of STAT3 axis

We utilized the STRING analysis tool (http://www.string-db.org/), a database of known and predicted protein interacting, to determine interacting proteins using STAT3 as the query [58].

### Statistical analysis

All statistical analysis was performed using GraphPad Prism version 5 (GraphPad Software, La Jolla, CA, USA). Survival curves were plotted using the cBioPortal and Kaplan–Meier plots. All results are displayed with *P* values from a long-rank test. Similarly, with Oncomine, heatmaps. A *P* values of < 0.05 were considered to be statistically significant.

## SUPPLEMENTARY MATERIALS FIGURES



## References

[1] Jing X, Cui X, Liang H, Hao C, Han C (2017). Diagnostic accuracy of ELISA for detecting serum Midkine in cancer patients. PloS one.

[2] Cui X, Jing X, Long C, Tian J, Zhu J (2017). Long noncoding RNA MEG3, a potential novel biomarker to predict the clinical outcome of cancer patients: a meta-analysis. Oncotarget.

[3] Salas LA, Johnson KC, Koestler DC, O'Sullivan DE, Christensen BC (2017). Integrative epigenetic and genetic pan-cancer somatic alteration portraits. Epigenetics.

[4] Xie S, Shen C, Tan M, Li M, Song X, Wang C (2017). Systematic analysis of gene expression alterations and clinical outcomes of adenylate cyclase-associated protein in cancer. Oncotarget.

[5] Ball MW, Gorin MA, Drake CG, Hammers HJ, Allaf ME (2017). The Landscape of Whole-genome Alterations and Pathologic Features in Genitourinary Malignancies: An Analysis of the Cancer Genome Atlas. European urology focus.

[6] Cui X, Liu J, Bai L, Tian J, Zhu J (2014). Interleukin-6 induces malignant transformation of rat mesenchymal stem cells in association with enhanced signaling of signal transducer and activator of transcription 3. Cancer science.

[7] Chang YC, Su CY, Chen MH, Chen WS, Chen CL, Hsiao M (2017). Secretory RAB GTPase 3C modulates IL6-STAT3 pathway to promote colon cancer metastasis and is associated with poor prognosis. Molecular cancer.

[8] Wu Z, Guo L, Ge J, Zhang Z, Wei H, Zhou Q (2017). Two serine residues of non-metastasis protein 23-H1 are critical in inhibiting signal transducer and activator of transcription 3 activity in human lung cancer cells. Oncology letters.

[9] Zhou J, Wu A, Yu X, Zhu J, Dai H (2017). SIRT6 inhibits growth of gastric cancer by inhibiting JAK2/STAT3 pathway. Oncology reports.

[10] Wang H, Deng J, Ren HY, Jia P, Zhang W, Li MQ, Li SW, Zhou QH (2017). STAT3 influences the characteristics of stem cells in cervical carcinoma. Oncology letters.

[11] Johnson M, O'Connell M, Walter K (2017). STAT3 activation and risk of recurrence in meningiomas. Oncology letters.

[12] Carpenter RL, Lo HW (2014). STAT3 Target Genes Relevant to Human Cancers. Cancers.

[13] Bredel M, Bredel C, Juric D, Harsh GR, Vogel H, Recht LD, Sikic BI (2005). Functional network analysis reveals extended gliomagenesis pathway maps and three novel MYC-interacting genes in human gliomas. Cancer research.

[14] Sun L, Hui AM, Su Q, Vortmeyer A, Kotliarov Y, Pastorino S, Passaniti A, Menon J, Walling J, Bailey R, Rosenblum M, Mikkelsen T, Fine HA (2006). Neuronal and glioma-derived stem cell factor induces angiogenesis within the brain. Cancer cell.

[15] Richardson AL, Wang ZC, De Nicolo A, Lu X, Brown M, Miron A, Liao X, Iglehart JD, Livingston DM, Ganesan S (2006). X chromosomal abnormalities in basal-like human breast cancer. Cancer cell.

[16] Finak G, Bertos N, Pepin F, Sadekova S, Souleimanova M, Zhao H, Chen H, Omeroglu G, Meterissian S, Omeroglu A, Hallett M, Park M (2008). Stromal gene expression predicts clinical outcome in breast cancer. Nature medicine.

[17] D’Errico M, de Rinaldis E, Blasi MF, Viti V, Falchetti M, Calcagnile A, Sera F, Saieva C, Ottini L, Palli D, Palombo F, Giuliani A, Dogliotti E (2009). Genome-wide expression profile of sporadic gastric cancers with microsatellite instability. European journal of cancer.

[18] Frierson HF, El-Naggar AK, Welsh JB, Sapinoso LM, Su AI, Cheng J, Saku T, Moskaluk CA, Hampton GM (2002). Large scale molecular analysis identifies genes with altered expression in salivary adenoid cystic carcinoma. The American journal of pathology.

[19] Pyeon D, Newton MA, Lambert PF, den Boon JA, Sengupta S, Marsit CJ, Woodworth CD, Connor JP, Haugen TH, Smith EM, Kelsey KT, Turek LP, Ahlquist P (2007). Fundamental differences in cell cycle deregulation in human papillomavirus-positive and human papillomavirus-negative head/neck and cervical cancers. Cancer research.

[20] Haferlach T, Kohlmann A, Wieczorek L, Basso G, Kronnie GT, Bene MC, De Vos J, Hernandez JM, Hofmann WK, Mills KI, Gilkes A, Chiaretti S, Shurtleff SA (2010). Clinical utility of microarray-based gene expression profiling in the diagnosis and subclassification of leukemia: report from the International Microarray Innovations in Leukemia Study Group. Journal of clinical oncology.

[21] Andersson A, Ritz C, Lindgren D, Eden P, Lassen C, Heldrup J, Olofsson T, Rade J, Fontes M, Porwit-Macdonald A, Behrendtz M, Hoglund M, Johansson B (2007). Microarray-based classification of a consecutive series of 121 childhood acute leukemias: prediction of leukemic and genetic subtype as well as of minimal residual disease status. Leukemia.

[22] Mas VR, Maluf DG, Archer KJ, Yanek K, Kong X, Kulik L, Freise CE, Olthoff KM, Ghobrial RM, McIver P, Fisher R (2009). Genes involved in viral carcinogenesis and tumor initiation in hepatitis C virus-induced hepatocellular carcinoma. Molecular medicine.

[23] Basso K, Margolin AA, Stolovitzky G, Klein U, Dalla-Favera R, Califano A (2005). Reverse engineering of regulatory networks in human B cells. Nature genetics.

[24] Saegesser F (1980). [Surgical treatment of pulmonary round foci detected in one male and eight female patients with breast cancer. Solitary metastasis, a second primary bronchopulmonary cancer or benign round foci? (author's transl)]. La semaine des hopitaux: organe fonde par l'Association d'enseignement medical des hopitaux de Paris.

[25] Zhan F, Barlogie B, Arzoumanian V, Huang Y, Williams DR, Hollmig K, Pineda-Roman M, Tricot G, van Rhee F, Zangari M, Dhodapkar M, Shaughnessy JD (2007). Gene-expression signature of benign monoclonal gammopathy evident in multiple myeloma is linked to good prognosis. Blood.

[26] Korkola JE, Houldsworth J, Chadalavada RS, Olshen AB, Dobrzynski D, Reuter VE, Bosl GJ, Chaganti RS (2006). Down-regulation of stem cell genes, including those in a 200-kb gene cluster at 12p13.31, is associated with *in vivo* differentiation of human male germ cell tumors. Cancer research.

[27] Gordon GJ, Rockwell GN, Jensen RV, Rheinwald JG, Glickman JN, Aronson JP, Pottorf BJ, Nitz MD, Richards WG, Sugarbaker DJ, Bueno R (2005). Identification of novel candidate oncogenes and tumor suppressors in malignant pleural mesothelioma using large-scale transcriptional profiling. The American journal of pathology.

[28] Barretina J, Taylor BS, Banerji S, Ramos AH, Lagos-Quintana M, Decarolis PL, Shah K, Socci ND, Weir BA, Ho A, Chiang DY, Reva B, Mermel CH (2010). Subtype-specific genomic alterations define new targets for soft-tissue sarcoma therapy. Nature genetics.

[29] Cenciarelli C, Marei HE, Felsani A, Casalbore P, Sica G, Puglisi MA, Cameron AJ, Olivi A, Mangiola A (2016). PDGFRalpha depletion attenuates glioblastoma stem cells features by modulation of STAT3, RB1 and multiple oncogenic signals. Oncotarget.

[30] Pei T, Meng Q, Han J, Sun H, Li L, Song R, Sun B, Pan S, Liang D, Liu L (2016). (-)-Oleocanthal inhibits growth and metastasis by blocking activation of STAT3 in human hepatocellular carcinoma. Oncotarget.

[31] Gabriele E, Brambilla D, Ricci C, Regazzoni L, Taguchi K, Ferri N, Asai A, Sparatore A (2017). New sulfurated derivatives of cinnamic acids and rosmaricine as inhibitors of STAT3 and NF-kappaB transcription factors. Journal of enzyme inhibition and medicinal chemistry.

[32] Dufait I, Van Valckenborgh E, Menu E, Escors D, De Ridder M, Breckpot K (2016). Signal transducer and activator of transcription 3 in myeloid-derived suppressor cells: an opportunity for cancer therapy. Oncotarget.

[33] Chen SF, Zhang ZY, Zhang JL (2017). Matrine increases the inhibitory effects of afatinib on H1975 cells via the IL6/JAK1/STAT3 signaling pathway. Molecular medicine reports.

[34] Huan W, Tianzhu Z, Yu L, Shumin W (2017). Effects of Ergosterol on COPD in Mice via JAK3/STAT3/NF-kappaB Pathway. Inflammation.

[35] Kaizu T, Ikeda A, Nakao A, Tsung A, Toyokawa H, Ueki S, Geller DA, Murase N (2008). Protection of transplant-induced hepatic ischemia/reperfusion injury with carbon monoxide via MEK/ERK1/2 pathway downregulation. American journal of physiology Gastrointestinal and liver physiology.

[36] Duan WN, Xia ZY, Liu M, Sun Q, Lei SQ, Wu XJ, Meng QT, Leng Y (2017). Protective effects of SOCS3 overexpression in high glucoseinduced lung epithelial cell injury through the JAK2/STAT3 pathway. Molecular medicine reports.

[37] Akinfenwa PY, Bond WS, Ildefonso CJ, Hurwitz MY, Hurwitz RL (2017). Versican G1 Domain Enhances Adenoviral-Mediated Transgene Expression and Can Be Modulated by Inhibitors of the Janus Kinase (JAK)/STAT and Src Family Kinase Pathways. The Journal of biological chemistry.

[38] Xu S, Zhao N, Hui L, Song M, Miao ZW, Jiang XJ (2016). MicroRNA-124-3p inhibits the growth and metastasis of nasopharyngeal carcinoma cells by targeting STAT3. Oncology reports.

[39] Cai X, Cao C, Li J, Chen F, Zhang S, Liu B, Zhang W, Zhang X, Ye L (2017). Inflammatory factor TNF-alpha promotes the growth of breast cancer via the positive feedback loop of TNFR1/NF-kappaB (and/or p38)/p-STAT3/HBXIP/TNFR1. Oncotarget.

[40] Liao XH, Xiang Y, Yu CX, Li JP, Li H, Nie Q, Hu P, Zhou J, Zhang TC (2017). STAT3 is required for MiR-17-5p-mediated sensitization to chemotherapy-induced apoptosis in breast cancer cells. Oncotarget.

[41] Gujam FJ, McMillan DC, Edwards J (2016). The relationship between total and phosphorylated STAT1 and STAT3 tumour cell expression, components of tumour microenvironment and survival in patients with invasive ductal breast cancer. Oncotarget.

[42] Liu X, Xiao Q, Bai X, Yu Z, Sun M, Zhao H, Mi X, Wang E, Yao W, Jin F, Zhao L, Ren J, Wei M (2014). Activation of STAT3 is involved in malignancy mediated by CXCL12-CXCR4 signaling in human breast cancer. Oncology reports.

[43] Klonowska K, Czubak K, Wojciechowska M, Handschuh L, Zmienko A, Figlerowicz M, Dams-Kozlowska H, Kozlowski P (2016). Oncogenomic portals for the visualization and analysis of genome-wide cancer data. Oncotarget.

[44] Zhou JJ, Cheng D, He XY, Meng Z, Li WZ, Chen RF (2017). Knockdown of Hotair suppresses proliferation and cell cycle progression in hepatocellular carcinoma cell by downregulating CCND1 expression. Molecular medicine reports.

[45] Liu XL, Zhang XT, Meng J, Zhang HF, Zhao Y, Li C, Sun Y, Mei QB, Zhang F, Zhang T (2017). ING5 knockdown enhances migration and invasion of lung cancer cells by inducing EMT via EGFR/PI3K/Akt and IL-6/STAT3 signaling pathways. Oncotarget.

[46] Luo S, Liu X, Zheng Y, Liu Y, Li Y, Wang W, Ni H, Liu Q (2015). Interleukin-22 inhibits tazarotene-induced gene 3 expression in HaCaT cells via MAPK-ERK1/2 and JAK2/STAT3 signaling. Journal of dermatological science.

[47] Atsaves V, Tsesmetzis N, Chioureas D, Kis L, Leventaki V, Drakos E, Panaretakis T, Grander D, Medeiros LJ, Young KH, Rassidakis GZ (2017). PD-L1 is commonly expressed and transcriptionally regulated by STAT3 and MYC in ALK-negative anaplastic large-cell lymphoma. Leukemia.

[48] Babaei Khalili M, Yazdanparast R, Nowrouzi A (2017). Induction of transient cell cycle arrest by H2 O2 via modulation of ultradian oscillations of Hes1, Socs3, and p-Stat3 in fibroblast cells. Journal of cellular biochemistry.

[49] Akinfenwa PY, Bond WS, Ildefonso CJ, Hurwitz MY, Hurwitz RL (2017). Versican G1 domain enhances adenoviral-mediated transgene expression and can be modulated by inhibitors of the Janus kinase (JAK)/STAT and Src family kinase pathways. The Journal of biological chemistry.

[50] Kitamura H, Ohno Y, Toyoshima Y, Ohtake J, Homma S, Kawamura H, Takahashi N, Taketomi A (2017). IL-6/STAT3 signaling as a promising target to improve the efficacy of cancer immunotherapy. Cancer science.

[51] Li Y, Cui N, Zheng PS, Yang WT (2017). BMX/Etk promotes cell proliferation and tumorigenicity of cervical cancer cells through PI3K/AKT/mTOR and STAT3 pathways. Oncotarget.

[52] Zhang Z, Wang A, Li H, Zhi H, Lu F (2016). STAT3-dependent TXNDC17 expression mediates Taxol resistance through inducing autophagy in human colorectal cancer cells. Gene.

[53] Rhodes DR, Yu J, Shanker K, Deshpande N, Varambally R, Ghosh D, Barrette T, Pandey A, Chinnaiyan AM (2004). ONCOMINE: a cancer microarray database and integrated data-mining platform. Neoplasia.

[54] Rhodes DR, Kalyana-Sundaram S, Mahavisno V, Varambally R, Yu J, Briggs BB, Barrette TR, Anstet MJ, Kincead-Beal C, Kulkarni P, Varambally S, Ghosh D, Chinnaiyan AM (2007). Oncomine 3.0: genes, pathways, and networks in a collection of 18,000 cancer gene expression profiles. Neoplasia.

[55] Cerami E, Gao J, Dogrusoz U, Gross BE, Sumer SO, Aksoy BA, Jacobsen A, Byrne CJ, Heuer ML, Larsson E, Antipin Y, Reva B, Goldberg AP (2012). The cBio cancer genomics portal: an open platform for exploring multidimensional cancer genomics data. Cancer discovery.

[56] Gao J, Aksoy BA, Dogrusoz U, Dresdner G, Gross B, Sumer SO, Sun Y, Jacobsen A, Sinha R, Larsson E, Cerami E, Sander C, Schultz N (2013). Integrative analysis of complex cancer genomics and clinical profiles using the cBioPortal. Science signaling.

[57] Gyorffy B, Surowiak P, Budczies J, Lanczky A (2013). Online survival analysis software to assess the prognostic value of biomarkers using transcriptomic data in non-small-cell lung cancer. PloS one.

[58] Szklarczyk D, Franceschini A, Wyder S, Forslund K, Heller D, Huerta-Cepas J, Simonovic M, Roth A, Santos A, Tsafou KP, Kuhn M, Bork P, Jensen LJ (2015). STRING v10: protein-protein interaction networks, integrated over the tree of life. Nucleic acids research.

[59] Talantov D, Mazumder A, Yu JX, Briggs T, Jiang Y, Backus J, Atkins D, Wang Y (2005). Novel genes associated with malignant melanoma but not benign melanocytic lesions. Clinical cancer research.

