# Supplementary material for: Systematic analysis of gene expression alterations and clinical outcomes of STAT3 in cancer
Source: Oncotarget. 2017 Dec 14;9(3):3198–213. doi: 10.18632/oncotarget.23226 (PMC5790457; doi:10.18632/oncotarget.23226)
Supplement: Supplementary file 1 [file oncotarget-09-3198-s001.pdf]

Systematic analysis of gene expression alterations and clinical outcomes of STAT3 in cancer

SUPPLEMENTARY MATERIALS

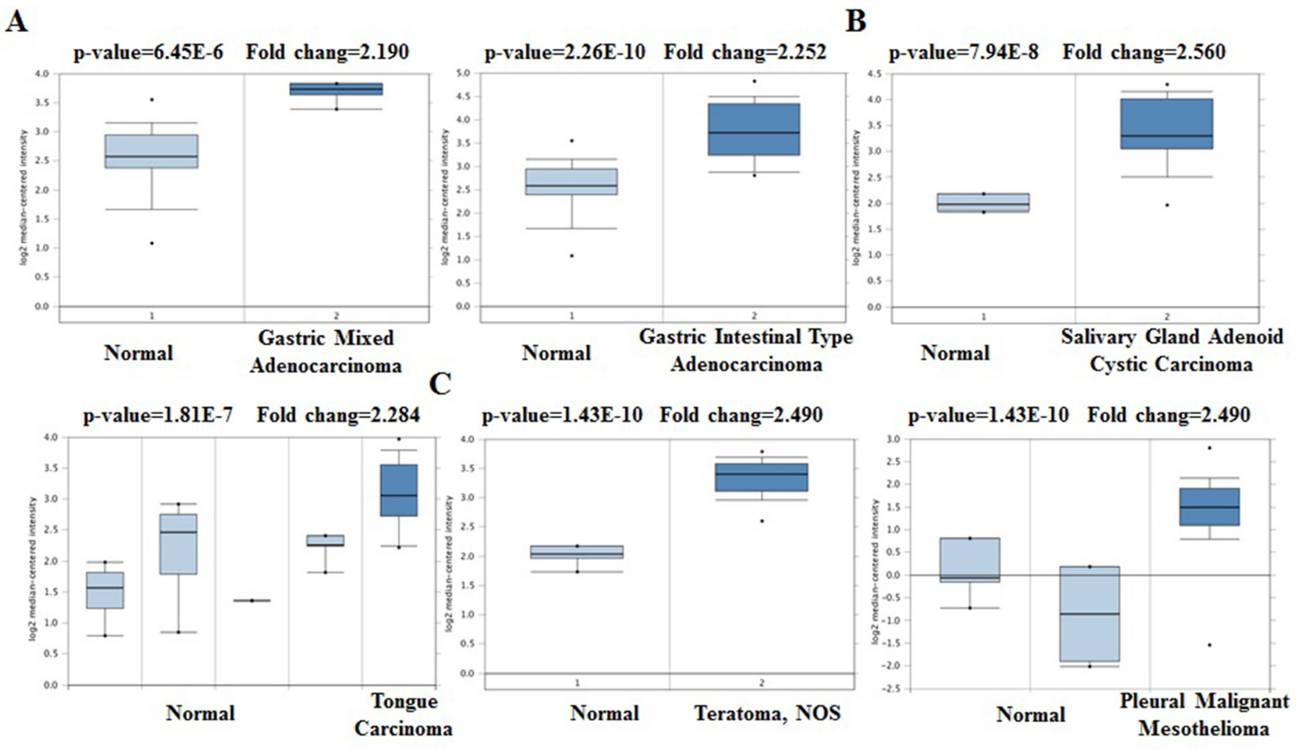

Supplementary Figure 1: The box plot comparing specific STAT3 expression in normal (left plot) and cancer tissue (right plot) was derived from Oncomine database. The analysis was shown in gastric adenocarcinoma relative to normal breast (**A**), in tongue and salivary gland adenoid cystic carcinoma to normal pancreatic (**B**), in teratoma and pleural malignant mesothelioma (**C**).

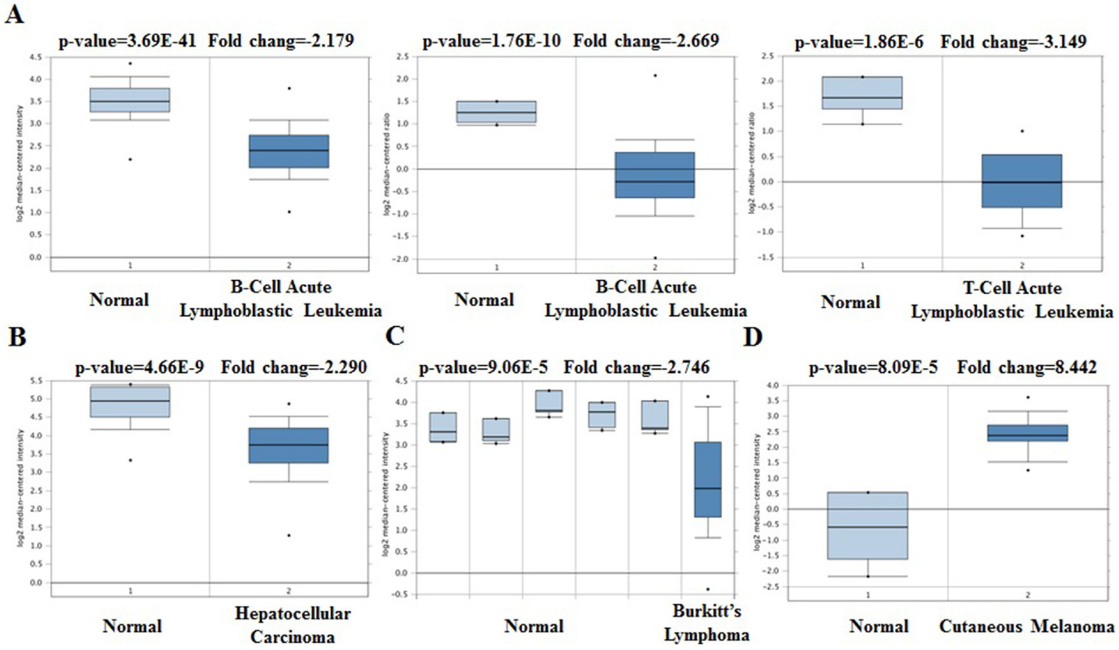

Supplementary Figure 2: The box plot comparing specific STAT3 expression in normal (left plot) and cancer tissue (right plot) was derived from Oncomine database. The analysis was shown in lymphoblastic leukemia relative to normal breast (**A**), in hepatocellular carcinoma to normal pancreatic (**B**), in burkitt's lymphoma (**C**), in cutaneous melanoma (**D**).

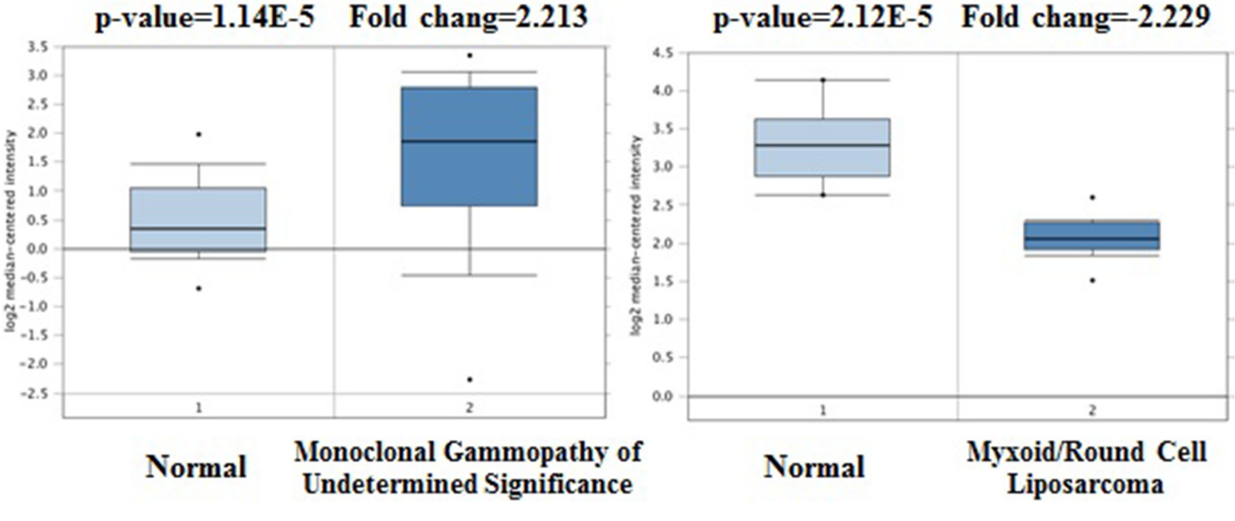

Supplementary Figure 3: The box plot comparing specific STAT3 expression in normal (left plot) and monoclonal gammopathy of undetermined significance and myxoid/round cell liposarcoma tissues (right plot) was derived from Oncomine database.
